# *In-Situ* Imaging of a Light-Induced Modification Process in Organo-Silica Films via Time-Domain Brillouin Scattering

**DOI:** 10.3390/nano12091600

**Published:** 2022-05-09

**Authors:** Sathyan Sandeep, Alexey S. Vishnevskiy, Samuel Raetz, Sergej Naumov, Dmitry S. Seregin, Artem Husiev, Konstantin A. Vorotilov, Vitalyi E. Gusev, Mikhail R. Baklanov

**Affiliations:** 1 Laboratoire d’Acoustique de l’Université du Mans (LAUM), UMR 6613, Institut d’Acoustique–Graduate School (IA-GS), CNRS, Le Mans Université, 72085 Le Mans, France; sandeep.sathyan@univ-lemans.fr (S.S.); samuel.raetz@univ-lemans.fr (S.R.); artemikg@ukr.net (A.H.); 2 MIREA—Russian Technological University, 119454 Moscow, Russia; alexeysw@mail.ru (A.S.V.); bxz1111@yandex.ru (D.S.S.); vorotilov@live.ru (K.A.V.); 3 Leibniz Institute of Surface Engineering (IOM), 04318 Leipzig, Germany; sergej.naumov@iom-leipzig.de; 4 European Centre for Knowledge and Technology Transfer (EUROTEX), 1040 Brussels, Belgium

**Keywords:** time-domain Brillouin scattering, two-photon absorption, low-k curing, mechanical properties

## Abstract

We applied time-domain Brillouin scattering (TDBS) for the characterization of porogen-based organosilicate glass (OGS) films deposited by spin-on-glass technology and cured under different conditions. Although the chemical composition and porosity measured by Fourier-transform infrared (FTIR) spectroscopy and ellipsometric porosimetry (EP) did not show significant differences between the films, remarkable differences between them were revealed by the temporal evolution of the Brillouin frequency (BF) shift of the probe light in the TDBS. The observed modification of the BF was a signature of the light-induced modification of the films in the process of the TDBS experiments. It correlated to the different amount of carbon residue in the samples, the use of ultraviolet (UV) femtosecond probe laser pulses in our optical setup, and their intensity. In fact, probe radiation with an optical wavelength of 356 nm appeared to be effective in removing carbon residue through single-photon absorption processes, while its two-photon absorption might have led to the breaking of Si-CH_3_ bonds in the OSG matrix. The quantum chemical calculations confirmed the latter possibility. This discovery demonstrates the possibility of local modifications of OSG films with a nanometric resolution via nonlinear optical processes, which could be important, among other applications, for the creation of active surface sites in the area-selective deposition of atomic layers.

## 1. Introduction

Quantitative and qualitative analysis of through-thickness inhomogeneity is extremely important during the development of functional materials for different applications. Time-domain Brillouin scattering (TDBS) is a rather new, all-optical experimental technique based on ultrafast lasers applied for the generation and detection of coherent acoustic pulses [1]. Pump laser pulses of a sub-picosecond to picosecond duration launch picosecond coherent acoustic pulses (CAPs) of a nanometer spatial length in the tested materials. In transparent materials, the scattering of the probe laser beam, due to the acousto-optic (photoelastic) effect, by laser-generated CAPs travelling through the sample allows for the imaging of sample inhomogeneity. By monitoring the reflected probe light, information on the time development of the CAP is obtained as it travels through the experimental sample. The transient optical reflectivity of the sample recorded by the probe beam, as the acoustic nanopulse propagates in space, contains information on the acoustical, optical, and acousto-optical parameters of the material under study in the spatial position of the CAP. In the collinear scattering geometry, the TDBS provides access to the product of the sound velocity and the optical refractive index of the sample. Therefore, important information related to the inhomogeneity of the density and mechanical properties of the materials can be obtained. Previously, we showed that TDBS is a very efficient technique for nanoscale imaging of porous low dielectric constant (low-k) films [2,3]. Particularly, it allowed us, for the first time, to extract the depth profiles of the optical refractive index and the longitudinal elastic modulus, with a depth resolution of several tens of nanometers, in partially cured low-k films containing an amount of remaining porogen residue. This is a unique opportunity to obtain information that is extremely important for practical applications.

The materials tested in this work are organosilica glass (OSG) films developed as low dielectric constant insulators for interconnects in advanced ULSI (ultra large scale integration) devices. They are used to insulate metal conductors and, together with low resistivity metal wires, improve the integrated circuit performance by reducing the signal propagation delay (resistive-capacitive delay, or RC delay) and crosstalk noise [4]. The introduction of porosity into OSG films allows for a reduction in the dielectric constant. The silica-like matrix of OSG dielectrics is formed from organosilane or alkoxysilane precursors [4]. The bridging oxygen atoms in silica matrix (≈20%) are replaced by terminal methyl groups to provide the hydrophobicity of these films. Hydrophobicity is important because of the high dielectric constant of adsorbed water (≈80). Porosity is normally generated by using sacrificial porogens that are co-deposited together with the matrix material. The porogens, which are usually organic polymers, are removed after deposition by thermal annealing that can also be assisted by UV light or electron beams [5].

Porous OSG films have poor mechanical properties compared to SiO_2_ because of the porosity and replacement of bridging oxygen atoms by terminal methyl groups. This issue poses a considerable challenge for their integration in on-chip interconnects. Therefore, the curing process must not only remove the porogen but also optimize the short-ranged bonding structure in a low-k matrix and improve its mechanical properties [6]. The curing process is also critical for other low-k properties. If the curing conditions are too aggressive (for instance, if the UV photon energy is higher than 6.5 eV), the organic polymer can form amorphous carbon-like residue (the so-called porogen residue), which causes an increased leakage current and degrades the reliability of the integrated circuits [7]. The aggressive removal of porogen can also partially reduce the concentration of CH_3_ groups bonded to Si atoms and increase the concentration of silicon dangling bonds (oxygen vacancies) that affects electrical properties [8]. Therefore, the precise analysis of the curing process and its mechanisms is extremely important.

**Figure 1:**
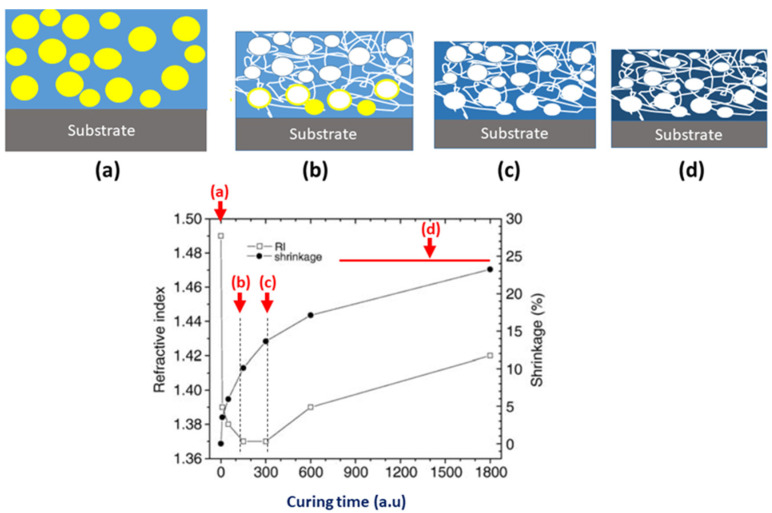
Changes in the low-k thickness and refractive index during the curing of a porogen-based low-k film. The curing curve is partially redrawn from [9]. The curing time is represented in arbitrary units because it depends on conditions. The region between the dashed curves corresponds to 1–2 h in pure thermal curing but can be reduced to 5–12 min if the curing is assisted by UV light as in [5]. The sketches in the subfigures (**a**–**d**) qualitatively visualize the state of the low-k film in the positions (**a**–**d**) indicated by the arrows in the main part of the figure. (**a**) The film before curing (soft baked film). (**a**,**b**) The removal of porogen causes the reduction in RI and thickness of the film. (**b**,**c**) The porogen is removed, but some residues can remain. (**c**,**d**) The matrix densification related to the dissociation of some Si-CH_3_ bonds and the collapse of a part of the nanopores causes increase in the RI.

An As-deposited film is a dense, two-component material containing a silica-like matrix and an organic porogen. The refractive index (RI) at 633 nm light is about 1.5–1.6 and depends on the properties of the matrix and the porogen. The post-deposition annealing removes the porogen and reduces the RI and the film thickness (left region in Figure 1a,b). The optimal curing time corresponds to the region between the two dashed lines (Figure 1b,c). In this region, the porogen molecules are almost or completely removed, but some residues can remain near the interface of the low-k film and the substrate [3,4,5]. The film in the region between Figure 1b,c) has the lowest index of refraction and dielectric constant and the highest porosity [5]. Further annealing (right region in Figure 1c,d) increases the RI because of the matrix densification related to the dissociation of some Si-CH_3_ bonds, the collapse of certain parts of the micropores and the molecular reorganization. The matrix densification in the (Figure 1c,d) region improves the mechanical properties but increases the dielectric constant and leakage current due to the reduction in CH_3_ group concentration and moisture adsorption. Therefore, the region where the porogen was almost fully removed (Figure 1b,c) is important for optimizing low-k material properties.

We used the TDBS technique for an evaluation of the curing efficiency of porous low-k films. Compared to our previous studies [2,3], when a low-k film was deposited on top of TaN (TaN is a standard diffusion barrier in Cu/low-k technology), the materials and structures in this work are more suited to the current strategy of replacing the damascene integration for a subtractive approach with alternative metals [10,11,12,13,14,15]. The main problems of the presently used Cu-based damascene technology with PECVD deposited low-k dielectrics are related to the increase in Cu resistivity in narrow lines [11], the degradation of low-k materials during the patterning in the etch plasma, and the need for diffusion barriers that have a much higher resistivity than Cu. All this increases the resistive-capacitive delay in signal propagation (RC-delay) and makes further ULSI interconnect scaling difficult [4]. Aluminum and its alloys [12,13] might also be possible alternatives to Cu due to the low resistivity in narrow lines and can be used without diffusion barriers between low-k dielectrics and metal. The subtractive integration consists of metal patterning and then filling the gaps with a flowable dielectric. The gap filling is normally based on an application of spin-on deposited dielectrics [14]. Subtractive integration with alternative metals without a diffusion barrier and gap-filling dielectrics will allow for a further scaling of IC interconnects for future technology nodes. In this new scenario, developing and understanding the low-k curing process is important and very challenging. It is necessary to mention that the list of alternative metals with a low resistivity in narrow lines also includes Ru, Co, Mo, etc. However, we selected Al in our research because of the deep knowledge of the opto-acoustic conversion processes in Al, following the absorption of ultrafast laser pulses [15]. Aluminum has a high optical reflectivity, enhancing the probe light scattered to the detector for the heterodyning of the weak probe light scattered by the acoustic waves. It also has high thermal conductivity, ensuring a low thermal loading of the low-k films in the TDBS experiments.

The aim of this research was to apply the TDBS technique to evaluate OSG low-k films cured in the critical region (Figure 1b,c) with different environments and curing times. The conducted TDBS experiments confirmed the existence of the differences between the films. More importantly, in the TDBS experiments on low-k films, we applied for the first time, UV (356 nm wavelength) femtosecond laser pulses as an ultrafast optical probe and revealed an additional curing of the low-k films, presumably caused by the their single-photon absorption by residual carbon and the breaking of the Si-CH_3_ bonds via their two-photon absorption. For TDBS depth-profiling, we applied a femtosecond pump-probe setup based on asynchronous optical sampling (ASOPS), which provided an opportunity to continuously follow the light-induced modifications of the low-k films with a 5 s temporal resolution. Our experimental observations point to the promise of using the two-photon absorption of UV laser pulses for the local modification of low-k materials with a nanometer spatial resolution.

## 2. Materials and Methods

### 2.1. Samples Preparation

The matrix precursor of OSG films was formed by the co-hydrolysis and co-condensation of tetraethoxysilane (TEOS–Si(OC_2_H_5_)_4_) and methyl-triethoxysilane (MTEOS–CH_3_Si(OC_2_H_5_)_3_) with a 3:2 mole ratio in a solvent mixture (70% isopropanol, ((CH_3_)_3_OH) and 30% ethanol (CH_3_CH_2_OH)). Hydrochloric acid, HCl, was used as a catalyst in accordance with the molar ratio [Si(OC_2_H_5_)_4_ + CH_3_Si(OC_2_H_5_)_3_]/H_2_O/HCl = 1/4/0.002. The solution was heated at 60 °C for 3 h under constant stirring. A surfactant (template) with a molar mass of 362 g/mol—Brij^®^ L4 (C_12_H_25_(OCH_2_OCH_2_)_4_OH) was added after cooling as a sacrificial porogen to produce the porous structure. The porogen concentration was kept equal to 19.3 wt. % relative to the total mass of metal alkoxide.

The films were spin-coated using a WS-650-8NPP (Laurell) spin coater at a rotation speed of 2500 rpm onto silicon wafers 100 mm diameter with an aluminum electrode (Si (111) 10 Ω/cm /Al 600 nm) and onto clean silicon wafers (Si (100) 12 Ω/cm). The wafers were then soft-baked on a hot plate at T = 200 °C for 30 min, and each one was cut into 4 pieces. The pieces were hard-baked in an oven at 400 °C for 60 and 120 min in air or in N_2_ to remove the organic residues and to complete polycondensation reactions (Table 1). The thickness and refractive indices (RIs) were measured on low-k films deposited directly on Si wafers. Table 1 shows that the RI slightly increased from the sample cured for 1 h in nitrogen (1 hN) to the sample cured for 2 h in air (2 hA). The films deposited on top of the Al interlayer were treated simultaneously with low-k films deposited directly on the Si substrate. It is reasonable to assume that the properties of low-k films deposited on top of Si and Al were completely identical.

The samples prepared for this research are methyl-terminated OSG. MTEOS precursor contains one methyl group bonded to Si and provides the introduction of methyl terminal groups. If the structure of pure SiO_2_ can be depicted as ≡Si-O-Si≡, where each Si atom is bonded to 4 oxygen atoms, the introduction of ≡Si-CH_3_ groups reduces the concentration of bridging oxygen atoms as well as the degree of interconnectivity of the Si backbone: ≡Si-CH_3_….H_3_C-Si≡.

### 2.2. Chemical Composition and Porosity

The chemical composition of the deposited films was analyzed by using low-k films deposited on top of Si and Fourier-transform infrared spectroscopy (FTIR). The transmittance spectra were recorded by using a Nicolet 6700 Fourier spectrometer (Thermo Fisher Scientific) with a resolution of 4 cm^−1^ and an extended wavenumber range between 7400 and 400 cm^−1^. A transmission mode with a resolution of 4 cm^−1^ was used with dry air purging. Background spectra were taken from a piece of pure silicon sample cut from the same wafer. For each sample, several (≈7–10) spectra were recorded with a relatively small (≈32) number of scans at different points and averaged to reduce the noise arising from changes in the atmospheric background. The baseline was corrected using a ~10th order polynomial function in combination with a sigmoidal function. The absorption intensity was normalized to the highest Si-O-Si peak.

The thickness and refractive indices of the films were measured by using a spectroscopic ellipsometer SENTECH 850 (300–850 nm). The open porosity and pore size were measured by using atmospheric pressure ellipsometric porosimetry. Isopropyl alcohol (IPA) vapors were diluted by nitrogen carrier gas and used as an adsorptive. The IPA partial pressure in nitrogen was controlled by using a specially designed thermostabilized bubbler. The open porosity *V* (the volume accessible for an adsorptive (*v_a_*) normalized to the film volume (*v_f_*)) was calculated by using an equation described in [16,17]:

(1) $$V=\frac{\left(\frac{n_{eff}^{2}-1}{n_{eff}^{2}+2}-\frac{n_{p}^{2}-1}{n_{p}^{2}+2}\right)}{\left(\frac{n_{ads}^{2}-1}{n_{ads}^{2}+2}\right)}$$

where *n_eff_* and *n_p_* are the refractive indices of the OSG film during the vapor condensation and of the film with empty pores (before adsorption), respectively, and *n_ads_* is the refractive index of the liquid adsorptive (*n_(IPA)_* = 1.377). The pore size was calculated from the curve of progressive pore filling (adsorption) and emptying (desorption) using Kelvin equation [17].

### 2.3. Time-Domain Brillouin Scattering Technique

**Figure 2:**
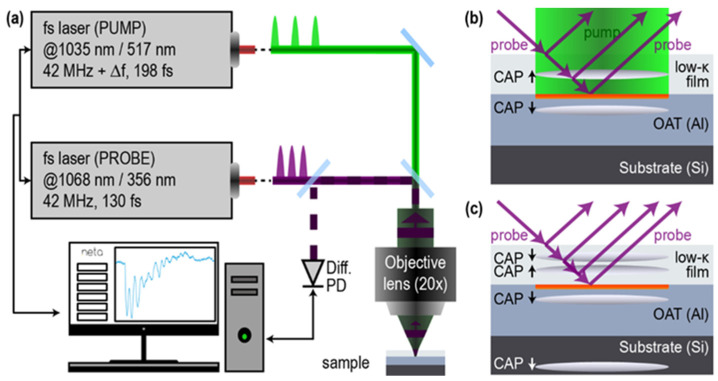
(**a**) Scheme of the experimental setup based on asynchronous optical sampling, where accumulating time delay between pump and probe femtosecond laser pulses is due to the difference in the repetition rate of two lasers. (**b**) Scheme of our TDBS experiment, where the absorption of the pump laser pulse in the Al film launches a coherent acoustic pulse (CAP) with a length of less than 25 nm in a low-k film and an Al layer. Scattering time-delayed probe light pulses by a CAP provides information on film properties in the current position of the CAP inside the film [2,3]. Particularly, the period of oscillating component in the presented time-domain transient optical reflectivity signals dR/R, as an inset in the screen of (**a**), is proportional to the product of local optical refractive index and sound velocity. (**c**) Illustration of the unwanted situation where the CAP propagating (**a**) in Al after partial reflection at Si/Al interface reaches the Al/low-k film interface and transmits a second CAP in the low-k film, which would scatter probe light simultaneously with the first CAP.

TDBS is an all-optical pump-probe technique based on the use of the high repetition rate femtosecond or picosecond laser pulses (Figure 2a). In application to our experimental low-k samples, its principle is illustrated in Figure 2b,c. The absorption of the pump laser pulse in an opaque optoacoustic (OA) transducer (an Al film on the Si substrate) launches a coherent acoustic pulse (CAP) of a nanometer scale length in the transparent low-k material. For example, the optical energy absorbed by the electrons in Al is transferred into an ultrafast Al lattice, heating up to a 50 nm depth from the surface [15]. The subsequent thermal expansion of Al launches into the low-k film the CAP of a characteristic length, which is about 2–2.5 times shorter than the characteristic depth of the optoacoustic conversion because of the slower velocity of the longitudinal acoustic waves in the low-k materials in comparison with Al ($v_{LA}^{Al}\approx 6.3–6.4\;$nm/ps, $v_{LA}^{low-k}\approx \left(2.4–3.2\right)$ nm/ps [2,3]). Thus, in the case of the Al OA transducer, the launched CAP does not exceed ≈8 ps in duration and ≈25 nm in length, when propagating in a low-k film. The time-delayed probe laser pulses are reflected at the free surface of the low-k film and at the interface between the low-k film and the OA transducer (see Figure 2). They are also scattered by the CAP due to the photo-elastic effect [18,19]. The strong reflected light and weak acoustically scattered light interfere at the photodetector, and the measured acoustically induced changes in the transient probe light reflectivity reveal the information on the parameters of the low-k film in the current position of the CAP [2,3]. When the CAP propagates in the materials with slowly varying inhomogeneity, the acoustically induced transient reflectivity changes have the form of a sinusoidal oscillation with a slowly but continuously varying amplitude and phase/frequency (see the examples of a measured signal as an inset in the screen of Figure 2a and in Figures 4 and 7 later in this article), which is known as Brillouin oscillation (BO) [1,20]. The quasi-sinusoidal oscillation of the acoustically induced probe light reflectivity in time is due to the quasi-linearly variation in the time phase shift on the photodetector between the light scattered by stationary surfaces/interfaces and the light scattered by the CAP moving at sound velocity. In the experiment geometry of Figure 2b,c, the frequency of the BO is equal to the Brillouin frequency shift in the 180° backward Brillouin scattering of light [1,18,19,20]:

(2) $$f_{B}=\left(2nv\right)/\lambda$$

where v is the longitudinal acoustic velocity in the medium, λ is the probe light wavelength in a vacuum, and n=n(λ) is the refractive index. The depth distributions of the refractive index, the sound velocity, and the efficiency of the photo-elastic interaction in sub-µm thick low-k films have been profiled by TDBS with a sub-100 nm spatial resolution [2,3]. This was achieved by conducting experiments at several angles of probe light incidence and applying dedicated and rather time-consuming signal processing to the detected BOs. In the research presented here, we were interested in the comparative characterization of differently prepared low-k films rather than in the depth profiling of their parameters. When the possible nanometer depth resolution is sacrificed in favor of simpler experiments (only a normal probe incidence in Figure 2) and less cumbersome signal processing, the information on the parameters of the low-k samples can be obtained by evaluating the averaged period and amplitude of the BOs. The larger the averaging time is, the lower the spatial resolution of the TDBS is, because the resolution would be limited not by the length of the CAP but by the length of the averaging interval. Here, we used the averaging over 1–2 periods of Brillouin oscillation. The length of 1 BO in the space domain is theoretically controlled by the wavelength of the probe light $\lambda_{probe}=\frac{\lambda }{n\left(\lambda \right)}$ in the transparent material because the acoustical wave, providing the most efficient backscattering of the probe light, has a wavelength that is twice as short as that of the probe light: $\lambda_{ac}=vf_{B}=\frac{\lambda_{probe}}{2}$, (Equation (2)). In fact, it is selected by the momentum conservation law in the photon–phonon interaction [1,19]. Thus, averaging over less than 2 BO leads to a spatial resolution that is better than $\lambda_{probe}=\frac{\lambda }{n\left(\lambda \right)}$, which is sub-optical.

### 2.4. Thermally and Acoustically Thick Optoacoustic Transducer for TDBS

In the research presented here, we were initially interested in a characterization of the low-k material near its interface with the Al film, and we did not intend to exploit the CAPs reflected from the free surface of the low-k film for this purpose (see Figure 2c for the time after CAP reflection). Therefore, although the goal was to characterize films up to 300–500 nm away from the Al/low-k film interface, the deposited films were thicker, i.e., about 600 nm (Table 1). We also deposited thick Al optoacoustic transducers, on the one hand, to diminish the temperature rise at its interface with low-k film and its influence on the low-k parameters and, on the other hand, to increase the depth of the “ideal” imaging. In fact, the photo-generated CAPs were launched not only in the low-k film but also in the Al film (see Figure 2b). When the CAP launched in Al after the reflection at the Al/Si interfaces returns to the Al/low-k interface and transmits into the low-k material, the TDBS is a result of two CAPs in the different spatial positions of the low-k film (see Figure 2c), making the extraction of the information on the parameters of the film more cumbersome. In our experiments, the Al films of a 600 nm thickness provided an opportunity to limit the maximum temperature rise at the interface due to the average heating by the pump and probe laser beams to less than 20 K and to monitor propagation in the low-k film of a single CAP up to about 500 nm away from the interface. The above estimate of the temperature rise was obtained from the available analytical formulas for the heating of a half space by the surface absorption of a Gaussian laser beam [21], assuming that the maximal pump and probe powers used in our experiments (Table 2) are both absorbed in Al but that the cooling of the surface is due to the transport of heat in the Si half space, i.e., a material with lower thermal conductivity than that of Al. In other words, the estimate assumed an infinitely small Al layer thickness in the thermal conduction problem, but it also assumed the absorption of the pump and probe light at the interface of the low-k film with bulk Al when estimating the light energy release in Al. 

However, we have verified with a more general theory [22] that accounting simultaneously for the actual finite thickness (≈600 nm) of Al film and the documented finite (not infinite) thermal conductivity across the Al/Si interface [23,24,25] does not modify the estimate of our experimental parameters listed in Table 2. The effect of the higher thermal conductivity of the Al film is practically compensated by the finite (not zero) thermal resistance at the Al/Si interface.

### 2.5. TDBS Setup Based on Asynchronous Optical Sampling (ASOPS)

The experimental setup was a commercial picosecond acoustic microscope (JAX-M1, NETA, Pessac, France) [26] based on asynchronous optical sampling (ASOPS). Two pulsed fiber lasers of fundamental optical wavelengths 1034.8 and 1068.4 nm, of pulse durations 198 and 130 fs, respectively, and with a repetition rate of about 42 MHz were synchronized for the ASOPS [27,28]. The repetition rate of the follower laser cavity (pump) was slightly offset compared to that of the leader one (probe). An offset of 500 Hz was used in our measurements, which corresponds to a temporal sampling of 0.28 ps over the approximately 24 ns (1/0.042 ns) duration of a recorded signal. The fundamental radiations were then doubled (pump) and tripled (probe) in frequency, leading to 517 nm for the pump beam and 356 nm for the probe beam. Note that, in Figure 2b,c, the probe beams were presented as obliquely incident for the convenience of the schematics, while both the pump and probe beams were actually normally incident and co-focused on the surface of the Al OAT. The spot size at the focal plane of the 20× objective lens was of an approximately 5 µm diameter at the 1/e^2^ level of the laser intensity. The averaged powers of the pump and probe lasers used in our experiments are summarized in Table 2.

The JAX-M1 picosecond acoustic microscope can be used to scan the pump beam relative to the probe beam in the focal plane of the objective lens, and this was done to assure a perfect overlap between both beams. Thanks to the ASOPS, which automatically offsets the arrival time of the probe pulse at the OAT compared to the arrival time of the pump pulse by a multiple of 0.28 ps, one measurement was accomplished within 2 ms. Because of this fast acquisition, there was no need to use a lock-in amplifier, as is usually done in pump-probe experiments using a mechanical delay line, and the signal-to-noise ratio of the experimental results presented in Section 3.3 was simply improved by averaging 1000 times, providing a temporal resolution with respect to the transient process dynamic of 5 s (accounting for transfer time from the acquisition card to the computer hard disk). It should be mentioned that it was possible to further reduce the acquisition time by a combination of the following possibilities: an increase in pump and probe pulse durations up to 10 times to minimize the peak power (hence allowing for higher powers), an increase in the amplitudes of the spectral components of the pump laser pulse intensity envelope at the frequencies of interest (a few tens of GHz), and/or changes in the repetition and the beating frequency of the ASOPS-based laser system. For instance, using a laser system with a 333 MHz repetition frequency and a beating frequency of 20 kHz, available commercially, would provide an opportunity to acquire signals with a duration of about 3 ns and a temporal sampling of 0.182 ps in 50 µs, hence reducing the temporal resolution for 1000 averages to about 50 ms, two orders of magnitudes better than our current setup.

### 2.6. Density Functional Theory

The mechanism of possible chemical transformations induced by UV photons was analyzed using density functional theory (DFT). The calculations were carried out systematically employing the PBE0-D3 density functional [29,30]. The way in which the PBE0 functional is derived and the lack of empirical parameters fitted to specific properties make the PBE0 model a widely applicable method for quantum chemical calculations. The PBE0-D3 functional includes physically and chemically important London dispersion interactions [31]. The molecular geometries, energies, and electronic structure of the molecules were studied at the PBE0-D3/6-31 G** level of theory, as implemented in the Jaguar 9.6 program [32]. This computational model was successfully used for calculations in our previous work [33,34,35]. Frequency calculations were performed at the same level of theory to obtain the total enthalpy (H) and Gibbs free energy (G). The reaction enthalpies (∆H) and Gibbs free energies of reaction (∆G) of the studied molecules were calculated as the difference of the calculated H and G between the reactants and products, respectively. The excited states and UV-Vis electronic transition spectra were calculated in the gas phase using the time-dependent (TD) DFT method [36] within the Tamm–Dancoff approximation [37] at the PBE0-D3/6-31 + G** level of theory. The Maestro 9.7 program [38] was used for the visualization of calculated UV-Vis spectra.

## 3. Results and Discussion

### 3.1. Chemical Composition

**Figure 3:**
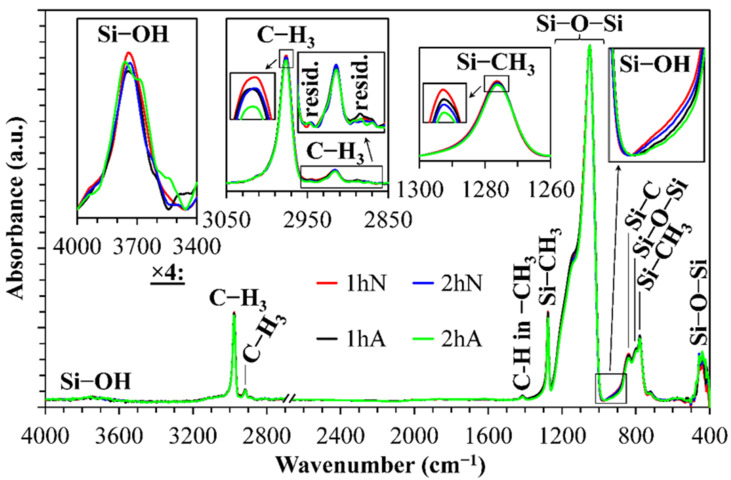
FTIR spectra of low-k films deposited and studied in this work: after hard-baking at 400 °C for 1 and 2 h in nitrogen (1 hN and 2 hN) and air (1 hA and 2 hA).

Figure 3 shows the FTIR spectra of the samples prepared for TDBS experiments. The curing conditions correspond to the minimum refractive index (Figure 1 and Table 1) when porogen was fully (or almost fully) removed. One can see that the chemical composition of all these films was very similar. 

The most pronounced peaks in the FTIR spectra are related to the silica-like matrix (Si-O-Si stretching vibrations at 1200–1000 cm^−1^) and to the terminal Si-CH_3_ groups (~1275 cm^−1^) and are typical for various OSG low-k films [4,5,9]. The concentration of the Si-CH_3_ groups was almost the same after different curing conditions, which suggests that all samples were cured near an optimal condition. Fully cured samples were free of the CH and CH_2_ bound fragments (about 2870 cm^−1^ and 2930 cm^−1^) that usually form from incompletely removed porogens. Some differences in the fully cured films can be seen in the peaks related to the C-H vibration at 2975 cm^−1^. This peak is mainly associated with the Si-CH_3_ groups. One can see that the samples cured for 1 h (1 hA) in air and 2 h in nitrogen (2 hN) were fully identical. The sample cured for 1 h in nitrogen (1 hN) had the highest carbon concentration, while the samples cured for 2 h in air (2 hA) had the lowest. These observations allow us to assume that the 1 hN sample contained some remaining carbon residues, while the 2 hA sample was completely cured. All studied samples contained a trace amount of silanol groups (Si-OH absorption near 3700 cm^−1^), but the difference between differently cured samples was very small.

All four films had similar porosity. The very small difference was related to the open porosity: 36.4% for the 1 hN and 1 hA samples and 36% and 35.9% for the 2 hN and 2 hA samples, respectively (Table 1).

### 3.2. TDBS Data

**Figure 4:**
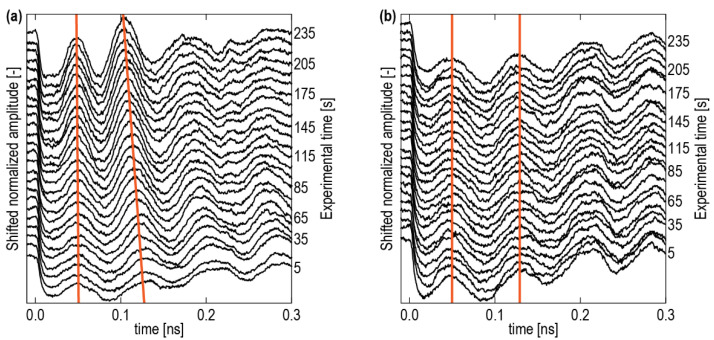
Vertically shifted normalized transient optical reflectivity signals ∆*R*/*R* recorded in the films (**a**) 1 hN and (**b**) 1 hA with a 26 mW pump power and a 1 mW probe power at different time points (right vertical axis) from the beginning of the TDBS experiment. Experimental time increases from the bottom to the top of each figure. The time interval along the horizontal axis between the two orange lines, passing the neighbor maxima of the Brillouin oscillations, provides an estimate of their period, i.e., the inverse of the Brillouin frequency (BF) in Equation (2). The revealed variation of the BF with experimental time in (**a**) is a signature of an additional curing of low-k films by laser light.

Figure 4 shows the typical optical reflectivity signals ∆*R*/*R*, accumulated over 5 s each, in the first 4 minutes of the TDBS experiments. The period of the so-called Brillouin oscillation, which was equal to the time interval between the two lines, diminished in the 1 hN film (Figure 4a), but it was not in the 1 hA film (Figure 4b). The 2 hN and 2 hA samples, for the same pump and probe laser powers, demonstrated a behavior that was similar to the 1 hA sample. The decrease in the interval between the two lines in the 1 hN sample was a signature of the continuous increase with the duration of the experiment regarding the product of the optical refractive index and the sound velocity.

**Figure 5:**
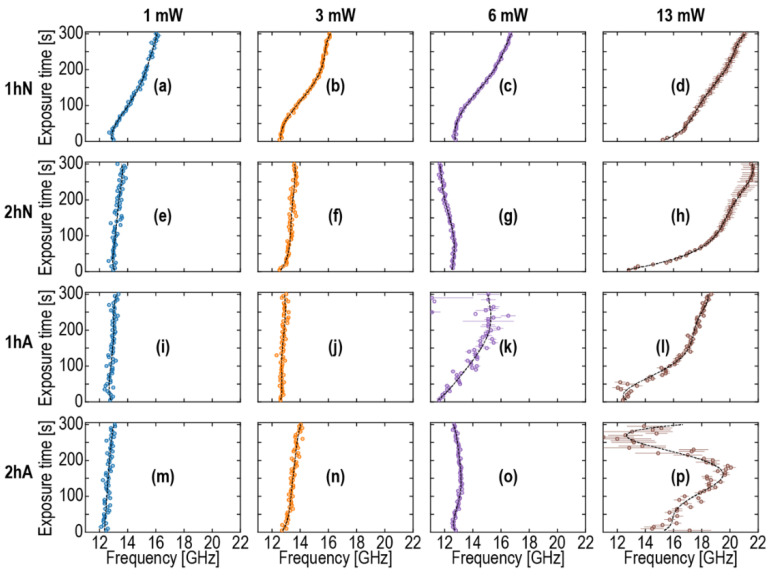
Changes in Brillouin frequency with the duration of the TDBS measurements at different powers of probe laser pulses (marked on the top of the figure) with a 26 mW pump power in the 1 hN (first row, (**a**–**d**)), 2 hN (second row, (**e**–**h**)), 1 hA (third row, (**i**–**l**)), and 2 hA (last row, (**m**–**p**)) samples.

**Figure 6:**
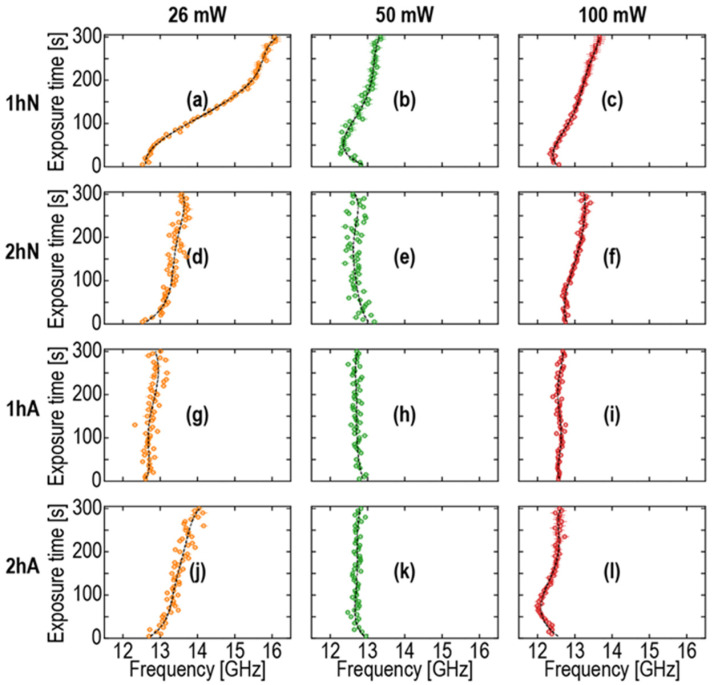
Changes in Brillouin frequency with the duration of the TDBS measurements at different powers of pump laser pulses (marked at the top of the figure) with a 3 mW probe power in the 1 hN (first row, (**a**–**c**)), 2 hN (second row, (**d**–**f**)), 1 hA (third row, (**g**–**i**)), and 2 hA (last row, (**j**–**l**)) samples.

The ∆*R*/*R* signals, similar to those presented in Figure 4, accumulated at different powers of pump and probe laser pulses, were used to estimate the variations of the Brillouin frequency with the experimental time in all four studied samples. The obtained results are shown in Figure 5 and Figure 6. To estimate the Brillouin frequency, the signal processing described in the following was applied in all cases except the 1 hA sample with a 6 mW probe power (Figure 5k) and the 2 hA sample with a 13 mW probe power (Figure 5p). First, an estimate of the Brillouin frequency was obtained by looking for the frequency at which a maximum of the power spectral density of the filtered signal occurs between 5 GHz and 30 GHz, where the used filter was a 24th-order Butterworth high-pass filter with a cut-off frequency of 5 GHz. Second, this estimated Brillouin frequency was used as the initial guess of a fitting routine, in which the raw signal between 0.02 ns and 0.18 ns was fitted by the model signal, $A\exp\left[-\alpha \left(t-t_{0}\right)\right]\cos\left[2\pi f_{B}\left(t-t_{0}\right)\right]+B+Ct+Dt^{2}$, where *A* is the amplitude of the damped cosine with damping factor α and (constant) frequency *f_B_*, *t*_0_ is a time shift, and the quadratic function with constants *B*, *C*, and *D* accounts for the slowly varying background. The initial guesses for all parameters were 0 for *t*_0_, *C*, and *D*, 0.1 for α, the mean value of the signal over the fitted time interval for *B*, and the difference between the maximum of the signal over the fitted time interval and *B* for *A*. *f_B_* is bounded between 5 and 30 GHz, α is bounded between 0 and 20 s^−1^, and the other parameters are unbounded. The fit was then obtained by minimizing the difference between the raw data and the model signal in the least mean square approach. When the convergence criterion was met, if the resulting value for the amplitude parameter *A* was very small (less than 10^−5^), the initial guesses of *A* and α would be changed by looking for closer-to-actual-values guesses, and the minimization would then be run once more. When the standard deviation error of the fitted Brillouin frequency was less than 1 GHz, the value was assumed trustworthy; otherwise, that value for the currently processed exposure time was not kept.

**Figure 7:**
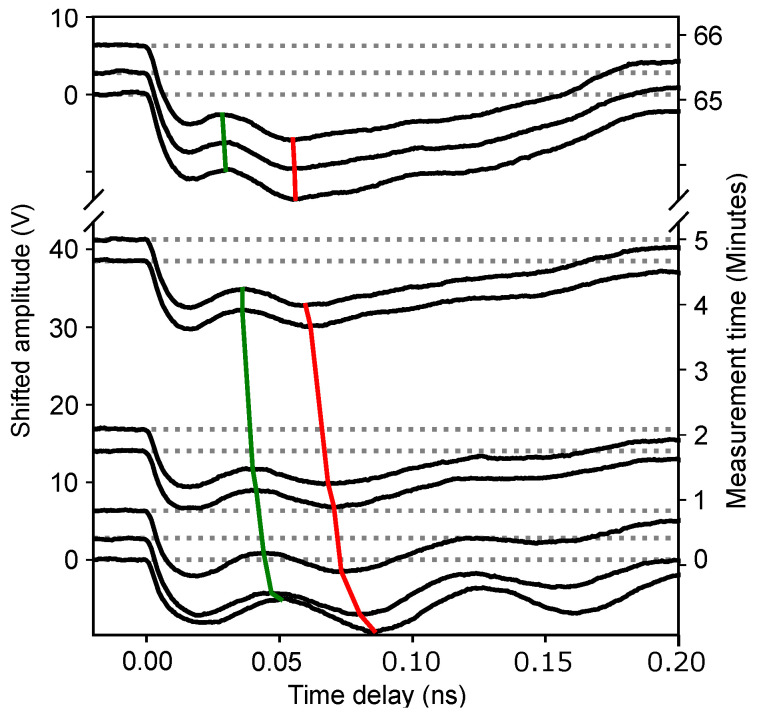
Irreversibility of the curing process demonstrated by the measurements of the acoustically induced transient optical reflectivity ∆*R*/*R* accompanied by the interruption of the experiments for 1 h after the first 5 min of the measurements. The green and red lines, passing through the maxima and minima of the first Brillouin oscillation, indicate a continuous diminishing of the Brillouin frequency with increasing experimental time. No increase in the Brillouin frequency was observed during the time when the experiment was stopped.

In the two “exceptional” cases (the 1 hA sample with a 6 mW probe power and the 2 hA sample with a 13 mW probe power), the Brillouin oscillations, after coherent acoustic pulse launching, vanished with time faster than in the other cases. This can be appreciated from the experimental results on the first of these samples, which are presented in Figure 7. While more than two Brillouin oscillations were clearly observed in the beginning of the TDBS experiment, less than one oscillation was distinguishable by the naked eye, i.e., without an application of signal processing, at the largest experimental time. In the “exceptional” cases, the end of the time interval for the fit was reduced from 0.18 to 0.1 ns. For both cases, the constraint on *f_B_* was more drastic: it was constrained to be ±6 GHz around the initial guess. For the 2 hA sample, the constraint on the parameter α was released to 50 s^−1^, while for the 1 hA sample, due to the atypical waveforms in the interval [0.02, 0.1] ns (see the signals in Figure 7 for the experimental times exceeding 4 min), the parameters α, *C*, and *D* were set and fixed to 0 for the fit. Thanks to this more appropriate signal processing, the BF was determined in the “exceptional” samples at the experimental times exceeding 200 s, yet with significantly larger uncertainty (less confidence) than in the other samples. We note here that, for the typical sound velocities of 2.8 nm/ps [2,3] in the low-k film, our fits conducted at times shorter than 0.18 ns after the CAP photo-induced generation correspond to the low-k films tested at distances shorter than 500 nm from the Al/low-k interface.

Figure 5a–d show the changes in Brillouin frequency in the 1 hN sample cured for 1 h in nitrogen. This was the softest curing condition, and according to FTIR spectra Figure 3, this sample contains the largest amount of carbon residue. It is reasonable to assume that this sample was not completely cured, and some carbon presented as a porogen residue (hydrocarbon species not bonded to Si). One can see that the Brillouin frequency measured in this sample changed when we used probe (356 nm) powers of 1 and 3 mW. This change became even more pronounced when the probe power was increased up to 13 mW. In all these cases, the pump (517 nm) power was equal to 26 mW. Additional experiments, when we used a constant probe power of 3 mW and different pump powers of 50 and 100 mW, showed that the effect of pump power was not significant, except in the 1 hN sample, especially at 26 mW (Figure 6a–c). Therefore, the change in Brillouin frequency was mainly defined by UV light with a 356 nm wavelength. This observation is in correlation with the maximum laser-induced temperature rise estimated in Section 2.4, 20 °K, which is significantly lower than the temperatures of the previous thermal curing (Table 1) and occurred at a much shorter TDBS experiment duration, 5 min, in comparison with the durations of the previous thermal curing (Table 1).

Figure 5e–p show the results of similar experiments when the curing conditions were more aggressive (2 h in nitrogen (2 hN), 1 h in air (1 hA), and 2 h in air (2 hA)). The curing in air is always more aggressive because the presence of oxygen helps to oxidize the porogen fragments and makes them volatile. One can see that, in all three cases, the Brillouin frequency did not change (in comparison to the 1 hN sample) when the probe powers were equal to 1 and 3 mW. At different pump powers and a constant 3 mW probe power, the Brillouin frequency variations in these samples (Figure 6) were also significantly smaller than in the 1 hN sample (Figure 6a–c). However, all three samples showed a significant change at a 13 mW probe power. Therefore, the carbon-rich 1 hN samples showed changes in Brillouin frequency at all used probe powers; other samples did not change at 1 and 3 mW, but Brillouin frequency changed at 13 mW of probe power.

For a better understanding of the observed phenomena, it is also important to analyze the reversibility of these changes. Figure 7 shows the results of the following TDBS experiment on the 1 hA sample. During the first 5 min, continuous measurements of the transient reflectivity signals were conducted at the same point on the sample with pump (λ=517 nm) and probe (λ=356 nm) powers of 26 and 6 mW, respectively. After pausing for 60 min, the experiments were repeated with the same parameters. The data acquisition time for a single measurement was 5 s; the intermediate signals are omitted in Figure 7 for clarity of presentation. The green and red lines pass through the maxima and minima of the first Brillouin oscillation, indicating the continuous diminishing of the Brillouin frequency with increasing experimental time. After a one-hour interruption of the experiment, the Brillouin frequency continued a slow diminishing. The absence of even a small increase in the Brillouin frequency caused by the interruption of the experiment reveals the irreversibility of the observed laser-induced curing process. Thus, the observed photo-induced changes in the chemical composition are irreversible.

### 3.3. Interpretation of TDBS Data: Origin of Brillouin Frequency Changes

As already mentioned, the Brillouin frequency is proportional to the product of the film’s index of refraction and sound velocity. Changes in the refractive index and sound velocity in low-k film can be associated with the densification of the matrix occurring due to the removal of remaining CHx residues from the matrix as well as with the improvement in cross-linking occurring after the removal of some parts of the CH_3_ terminal groups [5]. Therefore, for the discussion of the TDBS results, the low-k components that can be removed or modified under the used UV light will be considered. Analysis of FTIR spectra shows that the main groups in low-k films are associated with the presence of Si-O and Si-CH_3_ bonds in a low-k matrix and CHx residues embedded in the matrix or adsorbed on the pore wall. Energetic characteristics of these bonds are shown in Table 3.

One can see that the individual optical quanta used in our TDBS experiments were not able to cleave Si-O and Si-CH_3_ bonds. The shortening of the Brillouin oscillation period in the 1 hN film indicates that the Brillouin frequency was reduced by nearly 30% in 4 min, while other films (1 hA, 2 hN, and 2 hA) remained unmodified. This is related to the difference in the chemical composition of the 1 hN sample in comparison with the others. Indeed, it was originally expected by analyzing FTIR spectra that this sample obtained during the shortest curing time in a non-oxidizing atmosphere and therefore might contain some remaining hydrocarbon residues not bonded to silicon (template residuals, CHx) [40]. According to the available literature data [5], CHx species can absorb UV light with a 270–370 nm wavelength and could therefore undergo photochemical modification at the wavelength of our probe light (356 nm).

Interpretation of the TDBS results obtained on the other samples (1 hA, 2 hN, and 2 hA) is less obvious. These samples did not contain porogen residue, as indicated by FTIR analysis. This statement is supported by the fact that the Brillouin frequency did not change at probe powers of 1, 3, and 6 mW (Figure 5). The reason is that these films did not contain CHx residues, and the modification of SiCH_3_ and SiO bonds require much higher energy quanta than available. However, a Si-CH_3_ bond is energetically less stable than a Si-O bond, and a UV light with a wavelength ≤200 nm can break this bond through single photon absorption [41]. From quantum-chemical calculations on several model OSG substances, it was shown that a threshold wavelength necessary for the excitation of the ≡SiCH_3_ molecule into the first excited singlet state is about 190–200 nm. After excitation and inter-system crossing in an excited triplet state, scission of the Si–CH_3_ bond may occur. This finding reveals the presumption that only UV photons with a ≤190–200 nm wavelength can generate Si-centered radicals that subsequently attract protons from the neighboring methyl groups.

Alternative mechanisms need to be found to explain the observed changes in Brillouin frequency in all samples at the 13 mW probe power. We assumed that the most likely reason of the film modification was related to the two-photon absorption of the 356 nm UV light. The mechanism of this modification is presented in Section 3.4.

We also need to mention the sensitivity of the 1 hN film to the pump laser (519 nm) beam (Figure 6a–c). Again, these changes were observed only in the 1 hN sample, which according to the above discussions contains non-bonded hydrocarbon residues. It is also interesting that the changes were mostly observed at low power (26 mW). A simple possible explanation is that the photo-induced desorption of CHx hydrocarbons occurs at low power. At higher power, some parts of the hydrocarbon start to dissociate from photo-induced H-abstraction processes before desorption and form low volatile amorphous carbon-like residue [42]. This is the reason why efficiency of the carbon residue removal decreases at 50 and 100 mW of power.

### 3.4. Mechanism of Modification Based on Two-Photon Absorption

**Figure 8:**
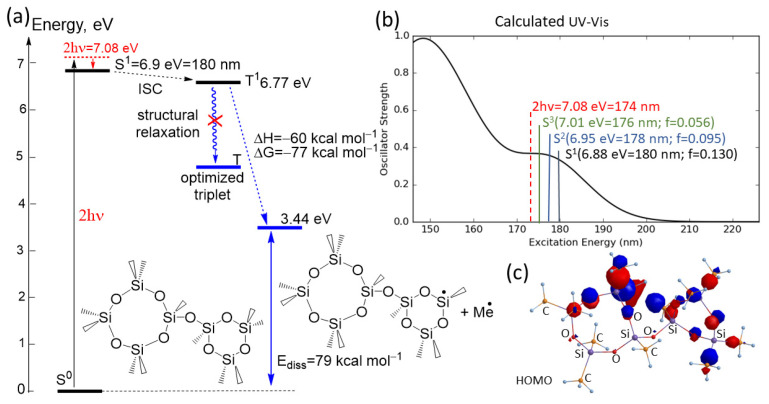
(**a**) Jablonski energy scheme of the population of the reactive triplet by two-photon excitation of the studied model molecule. (**b**) Calculated UV-Vis spectra; f-oscillator strength. (**c**) Electron distribution of σ-electrons from HOMO involved in the formation of the first S^1^ excited state.

To support the experimental findings, quantum chemical calculations were performed on the model molecule (Figure 8). The analysis of the formation of the reactive excited triplet state of the molecular fragment and the calculated UV-Vis spectra of the model molecule are shown in Figure 8a,b, respectively. The calculations show that the low energy absorption band with a maximum at 176 nm is formed through σ-electrons excitations from HOMO, HOMO−1, and HOMO−2, which are close in energy, into LUMO molecular orbitals. The distribution of σ-electrons from HOMO is shown in Figure 8c. The calculations were carried out in the gas phase, since the polymer was considered as a non-polar medium with a dielectric constant of about 2. To validate the used basis set, the calculated Si-O bond lengths were compared with X-ray data for Si-O lengths of various compounds presented by Baur [43]. The calculated average Si-O length values were only overestimated by about 3% compared to the X-ray data. It has also been shown [44] that the 6–31(d) basis set appears to be reliable for studying the structures and stabilities of silica materials. In our study, the higher basis set 6–31 + G(d,p) was used to calculate the excited states.

After two-photon absorption (350/2 nm = 2 × 3.54 eV= 7.08 eV), which should be sufficient for excitation to the S^1^, S^2^, and S^3^ states and following inner conversion (IC), the first excited singlet state S^1^ will be populated. After intersystem crossing (ISC), the triplet state T^1^ will be formed. This T^1^ will undergo further relaxation through adjustment of the molecular structure to the changes in electron distribution through the excitation. However, the structural relaxation of the excited T^1^ state into a more stable optimized triplet T does not occur. The excitation of electrons from HOMO leads to changes in atomic charge distribution in the molecule. Thus, the calculated Mulliken atomic charges on the Si and C atoms (1.09 and –0.65 in ground S^0^ state and 0.78 and –0.55 in T^1^ state) indicate a lower Coulomb attraction between Si and C atoms after excitation. Due to a strong weakening of the Si-C bond after excitation in the triplet state, the molecule is not stable and undergoes monomolecular Si-C bond scissions during optimization with the release of Me radical. This reaction is calculated as strong exergonic (reaction enthalpy ∆H = –60 kcal mol^−1^ and free Gibbs energy of reaction ∆G = –77 kcal mol^−1^).

To support these analysis, we have estimated that the peak intensities in our UV probe laser pulses (Table 2) varied from $5\times 10^{9}$ to $7\times 10^{10}$ W/m^2^. They were of the same order of magnitude as those that were sufficient to induce, in the experiments, the femtosecond multiphoton ionization and/or fragmentation of molecules [45,46] (at 627 and 510–540 nm optical wavelengths, respectively) and to measure the two-photon absorption in silicon carbide (SiC) [47,48] (at 800 and 400 nm optical wavelengths, respectively). This comparison makes our hypothesis on the nature of the observed low-k modifications even more plausible. At the same time, even the maximal peak intensities in our experiments were much lower than those corresponding to the damage threshold associated with multiphoton processes in silica (above 10^13^ W/m^2^ for the wavelengths from 266 to 800 nm [49]). Therefore, the two-photon absorption of 356 nm femtosecond laser pulses was able to modify the Si-CH_3_ bonds in the low-k film without damaging the silica matrix. This reveals the potential opportunity of using the two-photon absorption of UV laser pulses for the local curing of low-k materials with a nanometer spatial resolution based on the spatial confinement features of nonlinear optical processes, from which the laser machining and photo-polymerization of materials already benefit [50,51].

The possibility of such modifications reveal new, unexpected routes for nanoscale patterning, including area-selective modification, which is extremely important for novel patterning schemes based on self-alignment improvement [52]. Area-selective deposition is normally associated with the local generation of active surface sites for the atomic layer deposition (ALD) of necessary substances. In our case, this modification includes the selective removal of methyl groups from the low-k surface through two-photon chemistry with a femtosecond laser beam with a 356 nm wavelength. The generated CH_3_-free surface areas generated with nanometric resolution can react with water molecules generating surface silanols that are active sites for many different ALD processes.

## 4. Conclusions

Porous organosilicate low-k films were deposited by using spin-on glass technology. The films were thermally cured at 400 °C in air and nitrogen in the critical region when they had maximum porosity and the lowest dielectric function after complete porogen removal. This region is crucial for low-k properties because of the matrix re-arrangement leading to the highest quality of low-k films. The chemical composition measured by FTIR spectroscopy and porosity measured by ellipsometric porosimetry did not show significant differences between the films cured at different times.

Clearer differences were observed using time-domain Brillouin scattering. It was shown that the film cured for 1 h in nitrogen still had remaining porogen residue, as shown by the change in the Brillouin frequency of the reflected light. The porogen residue was removed by UV light with a 356 nm wavelength. The samples cured for 1 h in air and 2 h in nitrogen showed a stability at a 1–6 mW laser power, but Brillouin frequency changed at a 13 mW laser power, breaking Si-CH_3_ bonds. The latter fact was surprising, since it was previously shown that only more energetic UV photons with a wavelength shorter than 200 nm have sufficient energy to break Si-CH_3_ bonds. Quantum mechanical calculations were carried out and showed an ability to break these bonds at the high intensity of the femtosecond probe laser pulses due to two-photon absorption. This conclusion is important for understanding the UV light-based modification of low-k materials and reveals new possible routes for the generation of active surface sites for bottom-up nanofabrication by area-selective atomic layer deposition [53].

In brief, our research results demonstrate that time-domain Brillouin scattering can provide unique information that cannot be obtained by other experimental instrumentations. In our previous research [9,13], the possibility of evaluating the gradients of low-k properties at a nm resolution was demonstrated. In this work, we were able to detect extremely small differences in films cured in the critical region, when porogen had been almost completely removed, but the matrix formation had not yet been finished. The obtained results also demonstrated the local modifications of low-k films based on two-photon absorption. This discovery could be important for the development of active surface sites for area-selective ALD processes. It is important that the film modification process can be monitored in situ using time-domain Brillouin scattering.

## Figures and Tables

**Table 1:** Description of OSG samples with different curing times in nitrogen (N) and air (A). All characteristics presented in Table 1 were measured on witness Si samples without an Al interlayer. All samples were soft-baked at 200 °C for 30 min and then hard-baked (HB) at 400 °C.

Sample Number	HB Time (h)	d (nm) (±6)	RI (±0.0008)	Porosity (% ±0.2)	G (GPa) (±0.5)
1 hN	1	656	1.2586	36.4	3.6
2 hN	2	587	1.2601	36.4	3.0
1 hA	1	589	1.2611	36.0	3.0
2 hA	2	575	1.2621	35.9	2.6

**Table 2:** Parameters of the experimental setup applied for TDBS characterization.

ASOPS System	JAX-M1 (NETA, Pessac, France)
Probe repetition frequency	42 MHz
Pump repetition frequency	42.0005 MHz
Probe wavelength	356 nm
Pump wavelength	517 nm
Pulse duration	<200 fs
Beam diameter (1/e^2^)	5 µm (objective lens of 20× magnification)
Number of averages	1000
Acquisition time for 1000 averages	5 s
Probe power	1 mW, 3 mW, 6 mW, 13 mW
Pump power	26 mW, 50 mW, 100 mW

**Table 3:** The cleavage energy of low-k components [5,39].

OSG Fragments	Si-O-Si	Si-CH_3_	CHx
Energy of cleavage	>8.3 eV	>6 eV	3.4 eV
UV wavelength	<150 nm	<200 nm	365 nm

## Data Availability

Data underlying the results presented in this paper can be obtained from the authors upon reasonable request.
